# Incidentally Detected Lymphatic Filariasis in a Renal Allograft Recipient

**Published:** 2013-08-01

**Authors:** A. V. Vanikar, K. S. Suthar, V. B. Kute, S. J. Rizvi, H. L. Trivedi

**Affiliations:** 1 *Department of Pathology, Laboratory Medicine, Transfusion Services and Immunohematology, *; 2 *Department of Nephrology and Clinical Transplantation, *; 3 *Department of Urology and Transplantation, Dr HL Trivedi Institute of Transplantation Sciences (IKDRC-ITS), Ahmedabad, India.*

**Keywords:** Lymphatic filariasis, renal transplantation, lymphocele

## Abstract

Post-transplntation lymphocele is a well known complication, and lymphatic filariasis (LF) has occasionally been found to present as post-transplantation lymphocele. However, incidentally detected LF during transplantation surgery has not been reported. We present an incidentally detected LF presenting as enlarged lymph node in the right iliac fossa of a recipient during transplantation of donor kidney. He was subsequently treated after transplantation and had stable graft function without any complications after 8 months of follow-up.

## INTRODUCTION

Lymphatic filariasis (LF) is usually a disfiguring disease acquired in childhood and manifesting in adulthood with lymphedema of the legs or genital regions and with secondary bacterial infections. This vector-borne disease has been reported in endemic zones of India and even has been occasionally transmitted from donors [1, 2]. However an incidentally detected LF in a renal allograft recipient during transplantation surgery has not been reported yet.

## CASE REPORT

**Figure 1:**
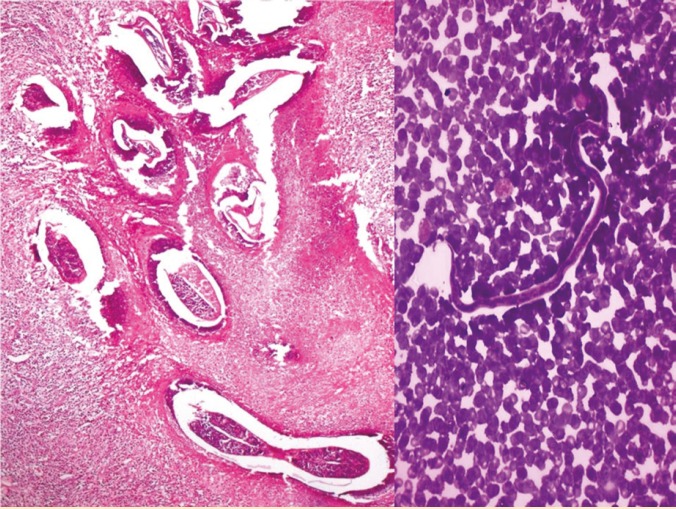
Left) Photomicrograph of the lymph node revealing necrotic lymphatic filled with coiled adult worms of microfilaria (H&E, ×100); Right) Peripheral blood smear showing Wuchereria bancrofti (microfilarial) worm (Leishman stain, ×200).

A 30-year-old man from suburbs of the city with renal failure was admitted for renal transplantation (RTx) from his 36-year-old HLA-identical (6/6) brother in January 2012. He was subjected to open right nephrectomy due to persistent pyuria in March 2012. Histopathology revealed chronic tubulointerstitial nephritis leading to end-stage renal disease. He was cleared for transplantation in June 2012. Before placing the renal allograft in the right iliac fossa, an enlarged lymph node was found and sent for histopathology. This was followed by an uneventful transplantation. Histopathology of the lymph node revealed necrotic lymphatic filled with coiled adult worms of *Wuchereria bancrofti* (microfilaria) (Fig 1) with double uteri in female worms. Subsequently after diethylcarbamazine citrate (DEC) challenge test, his peripheral blood also showed microfilarial worms (Fig 1), however he never had tissue/peripheral blood eosinophilia. He was therefore treated with a single dose of 400 mg albendazole followed by DEC, 6 mg/kg body weight for 14 days. Eight months post-transplantation he was maintaining stable graft function with serum creatinine of 1 mg/dL on maintenance immunosuppression of 0.06 mg/kg/day of tacrolimus, 360 mg of mycofenolate sodium twice a day, and 10 mg/day prednisone. On screening, none of his family members had evidence of filariasis.

## DISCUSSION

LF is caused by thread-like worms of genus *Wuchereria *and *Brugia, *known as filariae, that lodge in the lymphatic system and is borne by culex mosquitoes [1]. It may remain clinically silent or present with lymphedema, malaise or acute fever. About 40% of patients have renal involvement with proteinuria and hematuria. The parasites are deposited on the skin at the entry site. Over 6 to 12 months, these larvae migrate to lymphatic vessels and develop into adult worms, causing damage and dilatation of the lymphatic vessels. The filariae live for several years in the human host. During this period, they produce millions of immature microfilariae that circulate in the peripheral blood and are ingested by mosquitoes when they bite the infected humans. The larval forms further develop inside the mosquito before becoming infectious to humans, thus establishing a cycle of transmission [3].

In renal allograft recipients, LF may be transmitted from donors [2, 4, 5]. It may present as lymphocele after transplantation. However, to the best of our knowledge, this is the first report of an incidentally detected LF during transplant surgery presenting as an enlarged lymph node in the right iliac fossa.

The recommended treatment for asymptomatic LF is a single dose of 400 mg of albendazole and 6 mg/kg of body weight of DEC for 14 days. This treatment has cleared LF in RTx followed by an uneventful follow-up [3]. Our patient also responded well to this treatment.

On screening, it was found that the family hailed from rural India which was an endemic area for filariasis; however this patient was born and grew up in the suburbs of a non-endemic zone. It is possible that in childhood, he might have travelled to his native village where he was infested with this parasite but remained asymptomatic. Interestingly, on intense screening, none of the family members had evidence of filariasis.

## Notes

**FINANCIAL SUPPORT**: None.
